# Increased screw backout with non–inlay design retrograde femoral nails

**DOI:** 10.1007/s00590-026-04983-9

**Published:** 2026-10-06

**Authors:** Benyamin Dadpey, Kevin Zhu, Noah Sward, Allen Dao, William Kent

**Affiliations:** University of California San Diego, San Diego, USA

**Keywords:** Distal femur fracture, Retrograde intramedullary nail, Screw backout, Polyethylene inlay, Interlocking screws

## Abstract

**Purpose:**

To compare the rate of distal interlocking screw backout between inlay-design and non-inlay design retrograde intramedullary femoral nails in the treatment of distal femur fractures.

**Methods:**

Adults with AO/OTA 33 distal femur fractures treated with a retrograde ultra-high molecular weight polyethylene (UHMWPE) inlay-design nail (RFNA; DePuy Synthes) or a non-inlay design nail (T2 SCN; Stryker) between January 2015 and June 2025 at a single Level I academic trauma center with a minimum follow-up of 3 months were retrospectively reviewed. The primary outcome was distal interlocking screw backout. Secondary outcomes included time to backout diagnosis, backout distance, and rate of symptomatic screw removal. All outcomes were compared between the inlay-design and the non-inlay design cohorts.

**Results:**

A total of 134 patients were included: 76 in the inlay cohort and 58 in the non-inlay cohort. The inlay cohort demonstrated a significantly lower rate of distal interlocking screw backout compared to the non-inlay cohort (5.3% vs. 19.0%; RR 0.28, 95% CI 0.09–0.83; *p* = 0.02), corresponding to a 72.2% relative risk reduction. Time to backout diagnosis and backout distance did not differ significantly between designs. The rate of symptomatic screw removal was significantly lower in the inlay cohort (1.3% vs. 10.3%; *p* = 0.04). The distal transverse screw was the most common screw with backout in both cohorts.

**Conclusion:**

The ultra-high molecular weight polyethylene (UHMWPE) inlay at the distal interlocking interface of a retrograde femoral nail was associated with significantly lower rates of distal interlocking screw backout and symptomatic screw removal. When backout did occur, polyethylene inlay did not impact time to diagnosis and backout distance.

## Introduction

Distal femur fractures are uncommon orthopedic injuries, representing approximately 0.4% of all fractures and 3% to 6% of all femoral fractures with an incidence of 4.5/100,000. [1] The mainstay of treatment for distal femur fractures includes plate fixation, rIMN and nail-plate constructs. Retrograde IMN are typically used to treat extra-articular distal femur fractures or those supracondylar fractures with simple articular involvement. The theoretical benefits of rIMN for distal femur fractures are minimal soft tissue disruption and a load sharing construct to allow for early weight-bearing and mobility, which can result in a reduced postoperative morbidity. [2, 3]

Despite these advantages, using rIMN to treat distal femur fractures is not without limitations. Limited distal implant anchorage can affect angular and rotational stability of the construct which can lead to increased nail toggle and consequent distal interlocking screw loosening and backout with some studies reporting between 12 to 38% screw backout rate associated with older generation rIMNs [4–6]. To address these issues, a newer generation rIMN, Retrograde Femoral Nail Advanced (Depuy Synthes, West Chester, PA) has a ultra-high molecular weight polyethylene (UHMWPE) inlay at its distal nail-screw interface. This inlay was hypothesized to reduce micromotion at the screw-nail interface and therefore, reduce the rate of screw backout. The developmental tests have demonstrated that the inlay more than doubles the angular stability of the screws and reduces their toggle by 80% and increase resistance to pullout by 128% [7].

Despite this theoretical advantage, there is a wide range of screw backout rates reported in literature ranging from 9 to 38% when using inlay-design Retrograde Femoral Nail Advanced (RFNA) nail in treating distal and femoral shaft fractures. [7–13] Therefore, the goal of this study was to evaluate the impact of the inlay at the distal interlock of a retrograde intramedullary nail on distal interlocking screw backout by comparing the inlay-design nail with a non-inlay design T2 Supracondylar nail (SCN) (Stryker Trauma Mahwah, NJ) when used to treat distal femur fractures.

## Methods

### Patients

A retrospective review of all patients who underwent fixation of distal femur fractures at a Level I trauma center between January 1st, 2015, and June 30th, 2025, was conducted. Fractures were classified using the AO/OTA classification. Patients with AO/OTA 33 distal femur fractures were included if they (1) underwent retrograde intramedullary nailing of the femur with either a non-inlay design T2 SCN or a ultra-high molecular weight polyethylene (UHMWPE) inlay-design RFNA nail and (2) had at least three months of follow-up, as determined a priori. Patients were excluded from the study if they (1) were younger than 18 years of age, (2) had less than three months of follow-up, or (3) underwent retrograde nailing with implants other than T2 SCN or ultra-high molecular weight polyethylene (UHMWPE) inlay-design RFNA nails. The inlay in the RFNA nails used in this study were made from ultra-high molecular weight polyethylene (UHMWPE) and no UHMWPE inlay were swapped with a polyetheretherketone (PEEK) inlay. All non-inlay design nails were T2 Supracondylar Nails; the T2 Alpha Femur Retrograde Nailing System was not used, and no advanced locking bolts were implanted in any patient. The included patients were then divided into two groups: (1) patients treated with the non-inlay T2 SCN and (2) patients treated with inlay-design RFNA.

### Data collection

Electronic medical records were reviewed to obtain patients’ demographics including age, sex, body mass index (BMI), obesity, diabetes mellitus (DM), end stage renal disease (ESRD), coronary artery disease (CAD), peripheral artery disease (PAD), and anticoagulation status and social history including: alcohol, tobacco, marijuana, methamphetamine and opioid use along with fracture characteristics, fixation construct, postoperative weight-bearing restrictions, and implant complications including incidence of and time to screw backout, and rate of reoperation for screw removal. Radiographic and computed tomography images were reviewed to classify distal femur fractures using both AO/OTA classification and descriptively (supracondylar, intercondylar and intra-articular). Intraoperative fluoroscopic images and operative implant logs were used to identify implants. Postoperative radiographs were used to identify screw backout and backout distance.

### Outcomes

The primary outcome was incidence of screw backout rate defined as more than 1 mm of distal interlocking screw backout identified on any postoperative radiograph compared to the immediate postoperative radiographs, following the inclusion criteria that has been previously reported by Bhale et al. to include all screw backout potentially detectable by routine postoperative plain radiographs [10] Secondary outcomes included (1) time to screw backout, (2) distance backed out and (3) rate of symptomatic hardware removal.

### Statistical analysis

Statistical analysis was performed using IBM SPSS Statistics Version 30 (IBM Corp., Armonk, NY, USA). Normality of continuous variables was assessed using the Shapiro–Wilk test. Because continuous variables were not normally distributed, they were summarized as median [interquartile range] and compared using the Mann–Whitney U test. Categorical variables were summarized as counts and percentages and compared using Pearson chi-square or Fisher’s exact test, as appropriate. For variables with more than two categorical levels, overall comparisons were performed using Pearson chi-square testing, with post hoc pairwise comparisons and Bonferroni correction when indicated. Temporal trends in ordered categorical variables across the study period were assessed using the chi-square test for trend. Binary outcomes were additionally reported with exact binomial 95% confidence intervals where appropriate. Relative risks (RRs) with 95% confidence intervals were calculated for binary outcomes of interest to quantify the association between nail design and clinical outcomes. No missing data was found. All tests were two-sided. To estimate the association between nail design and distal interlocking screw backout independent of potential confounders, a multivariable logistic regression model was constructed including nail design, fracture classification (33C versus 33A), use of adjunctive fixation (lateral attachment washer, lateral plate and bolts) and postoperative weight-bearing status (weight bearing as tolerated versus restricted). Given the small number of backout events, the model was fitted using Firth penalized likelihood, with 95% confidence intervals derived from the profile likelihood. *P* < 0.05 was considered statistically significant.

## Results

### Patient and fracture characteristics

A total of 446 patients were identified who underwent rIMN for femur fractures with a non-inlay design nail or an inlay design nail. One hundred and thirty-four patients with AO/OTA 33 distal femur fractures with at least 3 months of follow-up were identified and included in this study. Fifty-eight patients underwent rIMN with a non-inlay design nail while 76 patients underwent rIMN with an inlay-design nail. Both cohorts were comparable for age, sex, BMI, past medical history such as obesity, diabetes, end stage renal disease, and social history such as alcohol, tobacco, marijuana, methamphetamine and opioid use. Patients in the inlay-design cohort had a significantly higher rate of CAD/PAD at baseline compared to patients in the non-inlay cohort (25% vs 10.3%, *p* = 0.03). The two cohorts were also comparable with respect to AO/OTA fracture classification, and rates of open, and periprosthetic fractures (Table 1).

**Table 1:** Patient demographics and fracture characteristics

		Non-Inlay (N = 58)	Inlay (N = 76)	*p*-Values
	Age	64 (47–74)	65 (55–76)	0.50
	Female sex	36 (62.1)	45 (59.2)	0.74
	BMI	28.6 (25.8–34.3)	29.4 (25.7–33.0)	0.81
Medical history	Obesity	25 (43.9)	36 (48.0)	0.64
	DM	21 (36.2)	29 (38.2)	0.82
	ESRD	4 (6.9)	6 (7.9)	1.00
	CAD/PAD	6 (10.3)	19 (25.0)	0.03^*^
	Anticoagulated	8 (13.8)	17 (22.4)	0.21
Social history	Alcohol use	10 (17.2)	10 (13.2)	0.51
	Active tobacco use	10 (17.2)	5 (6.6)	0.15
	Former tobacco use	9 (15.5)	13 (17.1)	
	Marijuana use	7 (12.1)	3 (3.9)	0.10
	Methamphetamine use	4 (6.9)	1 (1.3)	0.16
	Opioid use	2 (3.4)	0 (0.0)	0.19
Fracture characteristics	Pathologic fractures	2 (3.4)	2 (2.6)	1.00
	Open fracture	11 (19.0)	13 (17.1)	0.78
	Periprosthetic	21 (36.2)	23 (30.3)	0.48
AO/OTA classification	33A2	12 (20.7)	16 (21.1)	0.08
	33A3	21 (36.2)	35 (46.1)	
	33C1	1 (1.7)	7 (9.2)	
	33C2	13 (22.4)	13 (17.1)	
	33C3	11 (19.0)	5 (6.6)	

^a^Values are presented as n (%) for categorical variables and median (Interquartile range) for continuous variables

*p < 0.05 was considered statistically significant

### Operative treatment

The non-inlay design nail was used in isolation in 58.6% of patients. In 19% of patients, a condylar nut and screw were used at the distal interlocking portion of the nail. Plate nail fixation was used in 25.8% of patients in the non-inlay cohort.

In the inlay group, nail alone was used in 21.1% of patients. In 48.7% of patients a locking attachment washer was used with the RFNA. Plate nail fixation was used in 30.3% of patients in the inlay cohort.

Postoperatively, non-inlay nails had increased rate of weight bearing restrictions. Post hoc pairwise comparisons with Bonferroni correction showed a significantly higher proportion of non-weight-bearing patients with non-inlay design rIMN (70.7% vs 43.4%, *p* < 0.005), and a significantly higher proportion of weight bearing as tolerated restriction in patients with inlay-design nails (42.1% vs 15.5%, *p* < 0.005). Initial partial weight-bearing restrictions did not differ between groups (13.8% vs 14.5%, *p* = 0.911). Length of hospitalization was similar among both groups (Table 2).

**Table 2:** Surgical Indication for reduction, reduction construct, postoperative weightbearing restrictions and length of hospitalization

		Non-Inlay (N = 58)	Inlay (N = 76)	*p*-Values
Indication for surgery	Acute fracture	56 (96.6)	70 (92.1)	0.47
	Nonunion repair/revision surgery	2 (3.4)	6 (7.9)	
Reduction Construct	Nail	34 (58.6)	16 (21.1)	NA
	Nail and plate	13 (22.4)	23 (30.3)	
	Nail and LAW	NA	30 (39.5)	
	Nail, plate and LAW	NA	7 (9.2)	
	Nail and condyle nut and screw	11 (19.0)	NA	
Postoperative weight bearing Restrictions	NWB	41 (70.7)	33 (43.4)	0.005*
	PWB	8 (13.8)	11 (14.5)	
	WBAT	9 (15.5)	32 (42.1)	
	Length of hospitalization (days)	6 (4–10)	6 (4–9)	0.39

^a^LAW, locking attachment washer; NWB, non-weight bearing; PWB, partial weight bearing; WBAT, weight bearing as tolerated

^b^Values are presented as n (%) for categorical variables and median (Interquartile range) for continuous variables

*p < 0.05 was considered statistically significant. Post hoc pairwise comparisons with Bonferroni correction showed that postoperative weight bearing was restricted with NWB in higher proportion of non-inlay patients (70.7% vs 43.4%, *p* = 0.002), whereas postoperative weight bearing was restricted to WBAT in a higher proportion of Inlay patients (42.1% vs 15.5%, *p* = 0.001). Postoperative Partial weight bearing restriction did not differ between groups (13.8% vs 14.5%, *p* = 0.91)

Postoperative weight-bearing restrictions became progressively more permissive across the 10-year study period. During the first three years, when the T2 SCN was the only nail in use, weight bearing as tolerated was prescribed in 1 of 17 patients (5.9%) and non-weight bearing in 13 of 17 (76.5%). Over the fourth through sixth years, still with the T2 SCN, weight bearing as tolerated rose to 8 of 41 patients (19.5%) and non-weight bearing fell to 28 of 41 (68.3%). The RFNA replaced the T2 SCN at the start of the seventh year and was the only nail used thereafter. During the seventh through ninth years, weight bearing as tolerated was prescribed in 20 of 48 patients (41.7%) and non-weight bearing in 20 of 48 (41.7%); over the final eighteen months, weight bearing as tolerated was prescribed in 12 of 28 patients (42.9%) and non-weight bearing in 13 of 28 (46.4%) (*p* < 0.001 for the trend in weight bearing as tolerated and p = 0.004 for the trend in non-weight bearing, chi-square test for trend). Among patients treated with the nail alone, the same pattern was observed: weight bearing as tolerated was prescribed in 1 of 13 patients (7.7%) in the first three years and 4 of 21 (19.0%) in the fourth through sixth years with the T2 SCN, and in 6 of 12 (50.0%) in the seventh through ninth years and 2 of 4 (50.0%) in the final eighteen months with the RFNA (*p* = 0.011, chi-square test for trend).

### Distal interlocking screw backout

Median follow-up was comparable between the inlay and non-inlay cohorts (7.0 vs 6.3 months, respectively). With respect to the primary outcome, the rate of distal interlocking screw backout was significantly lower in the inlay cohort than in the non-inlay cohort (5.3%, 95% CI 1.5%–12.9% vs 19.0%, 95% CI 9.9%–31.4%), corresponding to a 72.2% relative reduction in screw backout risk associated with use of the inlay design (RR 0.28, 95% CI 0.09–0.83; *p* = 0.024).

Median time to diagnosis of screw backout was comparable between the inlay and non-inlay cohorts (2.4 months [IQR 1.3–3.5] vs 2.8 months [IQR 1.9–6.3], respectively). Median distal interlocking screw backout distance was greater in the non-inlay cohort compared to the inlay cohort (13.0 mm [IQR 9.5–18.0] vs 7.5 mm [IQR 4.5–11.5]), although this difference did not reach statistical significance (*p* = 0.19). The rate of symptomatic screw removal was lower in the inlay cohort than in the non-inlay cohort (1.3% [1/76] vs 10.3% [6/58]; *p* = 0.04).

On multivariable analysis adjusting for fracture classification, use of adjunctive fixation, and postoperative weight-bearing status, inlay design remained independently associated with a lower odds of distal interlocking screw backout (OR 0.20, 95% CI 0.05–0.71, *p* = 0.012). Neither fracture classification (OR 1.12, 95% CI 0.30–3.97, *p* = 0.86) nor adjunctive fixation (OR 0.77, 95% CI 0.24–2.46, *p* = 0.65) was associated with backout, while weight bearing as tolerated showed a non-significant trend toward higher odds of backout (OR 3.02, 95% CI 0.80–12.23, *p* = 0.10). The estimate for nail design was stable across model specifications, ranging from 0.18 to 0.27 with all covariate combinations tested.

Among the 11 distal interlocking screws that backed out in the non-inlay cohort, 10 (90.9%) were in the distal transverse position and one (9.1%) was in the proximal transverse position. Among the four distal interlocking screws that backed out in the inlay cohort, two (50.0%) were in the distal transverse position, one (25.0%) was in the proximal transverse position, and one (25.0%) was in the medial oblique position (Table 3).

**Table 3:** Distal interlock screw backout data

	Non-Inlay (N = 58)	Inlay (N = 76)	*p*-value
Follow-up (months)	6.3 (4.8–9.6)	7.0 (4.9–12.0)	0.55
Screw backout	11 (19.0, 9.9–31.4)	4 (5.3, 1.5–12.9)	0.02*
Distance backed out (mm)	13 (9.5–18)	7.5 (4.5–11.5)	0.19
Time to screw backout	2.8 (1.9–6.3)	2.4 (1.3–3.5)	0.27
Reoperation for screw removal	6 (10.3, 3.9 – 21.2)	1 (1.3, 0.03–7.1)	0.04*
Proximal transverse	1 (9.1)	1 (25)	NA
Distal transverse	10 (90.9)	2 (50)	
Medial oblique	0 (0)	1 (25)	
Lateral oblique	0 (0)	0 (0)	

^a^Values are presented as n (%) or n (%, 95% Confidence Interval) for categorical variables and median (Interquartile range) for continuous variables

*p < 0.05 was considered statistically significant

Exploratory comparison between patients with and without screw backout did not identify statistically significant differences in fracture characteristics, postoperative weight-bearing restrictions, or fixation constructs with inlay-design and non-inlay design nails (Table 4).

**Table 4:** Comparative of fracture classification, weight bearing restrictions and reduction construct in RFNA and T2 SCN groups with or without screw back out

		Non-Inlay design			Inlay design		
		No screw backout	Screw backout	*p*-value	No Screw Backout	Screw backout	*p*-Value
	Periprosthetic	16 (34)	5 (45.5)	0.50	22 (30.6)	1 (25)	0.99
AO/OTA	33A	26 (55.3)	7 (63.6)	0.74	48 (66.7)	3 (75)	0.99
	33C	21 (44.7)	4 (36.4)		24 (33.3)	1 (25)	
Weight bearing restrictions	WBAT	5 (10.6)	4 (36.4)	0.07	30 (41.7)	2 (50)	0.99
	NWB	34 (72.3)	7 (63.6)		31 (43.1)	2 (50)	
	PWB	8 (17.0)	0 (0)		11 (15.3)	0	
Construct	Nail only	27 (57.4)	7 (63.6)	0.99	15 (20.8)	1 (25)	0.99
	Nail with additional construct	20 (42.6)	4 (36.4)		57 (79.2)	3 (75)	

^a^NWB, non-weight bearing; PWB, partial weight bearing; WBAT, weight bearing as tolerated

^b^Values are presented as n (%)

### Patient characteristics and postoperative outcomes in patients treated with an inlay-design vs. non-inlay design nail in isolation

As presented in Tables 5 and 6, age, sex, BMI, medical history, fracture characteristics, AO/OTA classification and surgical indication were not statistically different between patients treated with inlay-design retrograde nail in isolation compared to those treated with non-inlay design retrograde femoral nails in isolation. Postoperative weight bearing restrictions were statistically different between groups with significantly higher weight bearing as tolerated restrictions in the inlay design group and a trend toward higher non-weight bearing restrictions in the non-inlay design group. While there was a trend toward lower screw back out rate and reoperating for screw removal in the inlay-design nail group compared to non-inlay design nail group, this difference did not reach statistical significance. Time to screw backout and distance backed out were not significantly different between groups. Lastly, distal transverse screw was the most common screw that experienced backout in both groups.

**Table 5:** Patient demographics and fracture characteristics in patients who received retrograde femoral nail in isolation

		Non-Inlay (N = 34)	Inlay (N = 16)	*p*-Value
	Age	62.0 (47.0–73.5)	51.0 (40.0–61.2)	0.17
	Female sex	19 (55.9)	8 (50.0)	0.70
	BMI	27.3 (25.1–34.2)	30.9 (26.1–33.1)	0.69
Medical history	Obesity	13 (39.4)	8 (53.3)	0.37
	DM	11 (32.4)	5 (31.2)	0.94
	ESRD	2 (5.9)	2 (12.5)	0.58
	CAD/PAD	4 (11.8)	3 (18.8)	0.67
	Anticoagulated	5 (14.7)	3 (18.8)	0.70
Social history	Alcohol use	6 (17.6)	3 (18.8)	1.00
	Active tobacco use	3 (8.8)	1 (6.2)	1.00
	Former tobacco use	6 (17.6)	1 (6.2)	0.41
	Marijuana use	6 (17.6)	1 (6.2)	0.41
	Methamphetamine use	4 (11.8)	0 (0.0)	0.29
	Opioid use	0 (0.0)	0 (0.0)	NA
Fracture characteristics	Pathologic fractures	1 (2.9)	0 (0.0)	1.00
	Open fracture	8 (23.5)	5 (31.2)	0.73
	Periprosthetic	13 (38.2)	2 (12.5)	0.10
AO/OTA classification	33A	23 (67.6)	10 (62.5)	0.72
	33C	11 (32.4)	6 (37.5)	0.72

^a^Values are presented as n (%) for categorical variables and median (Interquartile range) for continuous variables

*p < 0.05 was considered statistically significant

**Table 6:** Surgical Indication, postoperative weight-bearing restrictions, and distal interlocking screw backout data

		Non-Inlay (N = 34)	Inlay (N = 16)	*p*-Value
Indication for surgery	Acute fracture	33 (97.1)	13 (81.2)	0.09
	Nonunion repair/revision surgery	1 (2.9)	3 (18.8)	0.09
Postoperative weight bearing Restrictions	NWB	23 (67.6)	6 (37.5)	0.03*
	PWB	6 (17.6)	2 (12.5)	
	WBAT	5 (14.7)	8 (50.0)	
Outcomes	Follow-up (months)	5.4 (4.4–8.7)	7.0 (5.4–8.8)	0.49
	Screw backout	7 (20.6, 8.7–37.9)	1 (6.2, 0.2–30.2)	0.41
	Distance backed out (mm)	15.0 (11.0–20.5)	16.0 (n=1)	0.83
	Time to screw backout (months)	2.0 (1.7–4.4)	0.9 (n=1)	0.25
	Reoperation for screw removal	4 (11.8, 3.3–27.5)	1 (6.2, 0.2–30.2)	1.00
Screw position	Proximal transverse	2 (22.2)	0 (0.0)	NA
	Distal transverse	7 (77.8)	1 (100.0)	
	Medial oblique	0 (0.0)	0 (0.0)	
	Lateral oblique	0 (0.0)	0 (0.0)	

^a^NWB, non-weight bearing; PWB, partial weight bearing; WBAT, weight bearing as tolerated

^b^Values are presented as n (%) for categorical variables, n (%, 95% exact binomial confidence interval) for the primary and secondary binary outcomes, and median (interquartile range) for continuous variables

^c^Postoperative weight-bearing restriction was compared using an overall Pearson chi-square test across the three levels (*p* = 0.03). Post hoc pairwise comparisons with Bonferroni correction: NWB 0.044 (Bonferroni-adjusted 0.132); PWB 1.000 (Bonferroni-adjusted 1.000); WBAT 0.014 (Bonferroni-adjusted 0.043)

*p < 0.05 was considered statistically significant

## Discussion

This study investigated the role of the polyethylene inlay at the distal interlocking nail-screw interface of rIMNs on distal screw backout in the treatment of distal femur fractures. Compared to the non-inlay rIMNs, the inlay-design was associated with a significantly lower rate of screw backout and a significantly lower rate of symptomatic screw removal, suggesting a protective mechanical effect at the nail-screw interface. Although no statistically significant differences were observed in backout distance or time to diagnosis between cohorts, the reduction in both backout rate and subsequent surgical intervention supports the benefit of inlay design in distal interlocking constructs. Notably, regardless of implant design, the distal transverse screw remained the most susceptible screw to backout, occurring in 50% (2/4) of inlay and 91.1% (10/11) of non-inlay backout cases, highlighting this as a consistently high-risk screw for backout.

The screw backout rate observed in this study for the inlay-design (5.3%) is markedly lower than what has been reported in previous literature. In previous studies, distal interlocking screw backout rates with inlay design have ranged from 16.9 to 38% when treating AO/OTA 32 and 33 femur fractures [7–12]. Minhas et al. first drew attention to this problem, reporting inlay-design RFNA backout rates of 30% in treating diaphyseal and distal femur fractures [7]. Subsequent comparative studies reinforced this pattern: Bhale et al. reported a 24.5% backout rate with inlay-design RFNA versus 10.9% with non-inlay implants; Cintas et al. reported 23% versus 1.9% when comparing inlay-design RFNA to an older generation non-inlay nail; and Vlasak et al. reported backout rates of 25% and 7.14% for inlay and non-inlay designs respectively [8, 10, 12]. The highest rates were reported by Musick et al., who found a 38% overall backout rate with an inlay design, rising to 75% in metaphyseal fractures, compared to 5.1% with a non-inlay nail [9]. Piper et al., in a prospective cohort, reported a 16.9% backout rate for inlay-design RFNA and 4.7% retrospectively for non-inlay design T2 SCN [11]. Taken together, these studies establish a consistent benchmark of substantially elevated screw backout risk with inlay-design RFNA, making our observed rate of 5.3% a notable departure from the existing literature.

Several factors may account for the lower screw backout rate observed in our inlay cohort relative to prior studies. A key methodological distinction is that most published series restricted inclusion to patients treated with intramedullary nailing alone, whereas in our cohort, 21.1% of patients were treated with the nail in isolation. The remaining patients had additional fixation, in a form of either a locking attachment washer (LAW) or a supplemental plate, constructs that may confer additional rotational and angular stability at the distal segment and thereby attenuate the mechanical forces driving screw migration [14–16]. That said, subgroup analysis within our inlay cohort did not demonstrate a statistically significant difference in backout rate between patients treated with the nail alone versus those with adjunctive fixation.

A notable difference between cohorts was postoperative weight-bearing restriction. Patients in the non-inlay T2 SCN cohort were significantly more likely to be non-weight-bearing (70.7% vs. 43.4%), while patients in the inlay RFNA cohort were more frequently permitted weight-bearing as tolerated (42.1% vs. 15.5%). This likely reflects changing practice over time to encourage weight bearing as tolerated after fixation even with short distal segments. Postoperative weight-bearing restrictions became progressively more permissive over the 10-year study period, with weight bearing as tolerated increasing more than sevenfold and non-weight bearing declining correspondingly. Notably, this shift was already underway during the first six years, before the change in implant, with weight bearing as tolerated rising from 5.9 to 19.5% among patients treated with the non-inlay T2 SCN nails. The trend persisted when patients treated with additional fixation were excluded, indicating that it reflects an evolving approach to postoperative rehabilitation rather than a change in fixation strategy. This is further highlighted by the fact that the decision to switch from non-inlay to inlay technology at the institution where the study was conducted was not based on individual surgeon preference for a specific rIMN design, but rather on the year in which the institution changed its implant supplier. Difference in weightbearing restriction introduces a potential confounder: more permissive early loading in the inlay cohort would be expected to increase cyclic mechanical stress at the distal interlock, theoretically increasing and not reducing rates of screw backout. However, it is important to interpret this confounder in the context of the inlay-design cohort more commonly received fixation in addition to the nail, in the form of a locking attachment washer or plate. These constructs, increase rigidity at the distal nail segment and theoretically reduced micromotion, potentially offsetting the additional mechanical demands imposed by earlier weight-bearing [14–16]. In other words, the greater construct stability conferred by adjunctive fixation in the inlay cohort could offer an alternative explanation for why, despite more permissive postoperative loading, these patients experienced fewer screw backout events. While use of additional constructs and increased weight bearing as tolerated restriction in the inlay design cohort could potentially have confounded our findings, on multivariable analysis, inlay design remained independently associated with reduced screw backout after adjustment for fracture classification, adjunctive fixation, and weight-bearing status, and the use of adjunctive fixation was not itself associated with backout. Weight bearing as tolerated showed a non-significant trend toward higher odds of backout, consistent with the expected mechanical effect of earlier loading and indicating that this confounder, if anything, biased against rather than in favor of the inlay design. This suggests that the protective effect observed is attributable to the nail-screw interface rather than to construct rigidity or to differences in postoperative loading.

The median time to screw backout diagnosis was not significantly different between the inlay and non-inlay cohorts, suggesting that the polyethylene inlay, while protective against backout occurrence, does not alter the temporal pattern of screw migration once it begins. Compared to previous studies, time to initial screw backout was longer. Bhale et al. reported a median of 44 days, Cintas et al. 22 days, and Musick et al. 34 days postoperatively [9, 10, 12]. Our findings were more comparable to those of Minhas et al. at 61 days and Alcaide et al. at 73 days [7, 13]. This variability across studies likely reflects differences in institutional follow-up protocols and overall study duration rather than true biological differences in screw migration kinetics. Longer follow-up intervals between postoperative visits may delay radiographic detection of backout, particularly in cases where screw migration is gradual and initially asymptomatic.

The rate of symptomatic screw removal was significantly lower in the inlay cohort compared to the non-inlay cohort (1/4 [25%] (1/4 vs. 6/11 [54.5%]), a difference that may be partly explained by the magnitude of screw migration. Although it did not reach statistical significance, there was a trend toward shorter backout distance in the inlay cohort compared to the non-inlay cohort (7.5 mm vs. 13 mm), consistent with the hypothesis that the polyethylene inlay attenuates screw toggle and limits migration even when backout does occur. Screws that migrate a shorter distance are likely to remain less symptomatic, raising the threshold for surgical removal [4]. This finding extends the clinical relevance of the inlay beyond simply reducing backout incidence, suggesting it may also mitigate the severity of migration when it does occur. For comparison, Alcaide et al. reported a mean backout distance of 17 mm, more than twice that observed in our inlay cohort, alongside a removal rate of 55%, while Musick et al. and Minhas et al. reported removal rates of 55% and 63% respectively, all substantially higher than the 25% observed in our inlay cohort. [7, 9, 13]

The distal transverse screw was the most frequently observed screw backing out in both cohorts, accounting for 2/4 (50%) of backout events in the inlay group and 10/11 (90.9%) in the non-inlay group, identifying this screw position as a consistently high-risk location regardless of implant design. Hamaker et al. found that the most distal screw of the retrograde intramedullary femoral nail, particularly when placed less than 40 mm from the joint line, has the greatest tendency to irritate surrounding soft tissue upon migration, and therefore most frequently requires removal [4]. Given this established risk, routine use of an endcap, which compresses directly on the distal screw, may mechanically restrict its migration and warrants consideration as an adjunctive measure to further mitigate backout.

### Limitations

This study has several limitations that warrant consideration when interpreting its findings. First, its retrospective design introduces the potential for selection bias, unmeasured confounding, and dependence on the electronic medical record. Second, screw backout and prominence were assessed using plain radiographs rather than cross-sectional imaging, which may have underestimated the true magnitude of screw migration in some cases. Third, while the overall sample size was sufficient to detect a difference in the primary outcome, the low absolute number of backout events limited the statistical power of secondary analyses, including backout distance and time to diagnosis. Forth, there was a trend toward using more fixation in the form of lateral locking plate or locking attachment washer or both in the inlay-design cohort compared to non-inlay design cohort for surgical treatment of distal femur fractures which can potentially confound the impact of inlay on distal interlock screw back out by potentially providing additional fixation construct stability and reducing nail toggle. Fifth, with 15 backout events the multivariable model contains fewer than 10 events per variable; penalized estimation was used to mitigate small-sample bias, but the confidence intervals remain wide and the adjusted estimate should be considered supportive rather than definitive. Finally, patient-reported outcomes were not collected, precluding assessment of the clinical impact of radiographic screw backout on patient quality of life and recovery.

## Conclusion

The presence of an ultra-high molecular weight polyethylene (UHMWPE) inlay design at the distal interlocking interface of the retrograde femoral nail was associated with a 72% relative reduction in screw backout rate compared to a non-inlay design retrograde femoral nail (5.3% vs. 19%), and an 88% relative reduction in symptomatic screw removal rate (1.3% vs. 10.3%). This association persisted on multivariable analysis adjusting for fracture classification, adjunctive fixation, and postoperative weight-bearing status (OR 0.20, 95% CI 0.05–0.71). These findings suggest that the ultra-high molecular weight polyethylene (UHMWPE) inlay confers a protective mechanical effect at the nail-screw interface, attenuating both the incidence and severity of screw migration. Neither postoperative weight-bearing restriction nor the use of adjunctive fixation constructs appeared to independently influence backout rate. The distal transverse screw represented the highest-risk position for backout across both implant designs, and routine endcap use warrants consideration as a strategy to further reduce this complication.

## Data Availability

No datasets were generated or analysed during the current study.
